# Social skill training and children’s cognitive concentration in rural China: the mediating effect of social information processing skills

**DOI:** 10.3389/fpsyg.2025.1526065

**Published:** 2025-05-09

**Authors:** Na Li, Jin Peng, Xiaodong Sun, Shenyang Guo

**Affiliations:** ^1^School of Humanities and Social Sciences, Xi’an Jiaotong University, Xi’an, China; ^2^Brown School, Washington University in St. Louis, St. Louis, MO, United States

**Keywords:** social skills training, social information processing skills, cognitive concentration, mediating effect, rural China

## Abstract

**Background:**

The cognitive concentration refers to the learning orientation of children, encompassing the skills, attitudes, and behaviors exhibited by children in their pursuit of knowledge. Enhancing children’s cognitive concentration through targeted social skills training holds notable importance for improving their classroom performance and academic achievement.

**Methods:**

Drawing upon the social information processing (SIP) theory and the large-scale trial “Let’s Be Friends (Shaanxi)” program with a randomized controlled experimental design, we employed structural equation modeling (SEM) to analyze the impact mechanism through which social skills training influences children’s cognitive concentration and examine the mediating effect by SIP skills.

**Results:**

The findings revealed that social skills training significantly enhances both children’s SIP skills and cognitive concentration. SIP skills fully mediate the relationship between social skills training and children’s cognitive concentration. Furthermore, this mediating effect is more pronounced in enhancing boys’ cognitive concentration compared to girls.

**Conclusion:**

Social skills training programs focused on enhancing children’s SIP skills represents an effective strategy for augmenting their cognitive concentration.

## 1 Introduction

In recent decades, the social transformations and rapid advancements in China have considerably amplified the vulnerabilities associated with children’s development. These risk factors, operating at environmental, structural, and individual levels, contribute to deviations in children’s behavior pertaining to social interaction, participation, and academic performance (Wu et al., 2016). Among these factors, children’s learning performance is closely intertwined with their academic achievement and has garnered considerable attention. Deviations in children’s learning behavior can be assessed through cognitive concentration, which refers to the learning orientation of children, encompassing the skills, attitudes, and behaviors exhibited by children in their pursuit of knowledge, and it’s indicative of childhood aggression (Macgowan et al., 2002). Low cognitive concentration indicates inadequate adaptability to classroom and learning activities, characterized by absent-mindedness or easy distractibility during class as well as a lack of effort in studying that ultimately leads to diminished academic accomplishments. Previous studies have demonstrated that poor academic performance serves as a pivotal indicator for predicting negative developmental trajectories among children (Macgowan et al., 2002). Therefore, reinforcing children’s cognitive concentration is of paramount importance in fostering their holistic development across the life course.

Early identification and intervention for children displaying aggressive behavior can effectively mitigate the escalation of behavioral problems (Hawkins et al., 2005). However, addressing children’s behavioral problems within the context of environmental and structural factors poses considerable challenges (Wu et al., 2016). Consequently, scholars in psychology have sought to analyze aggressive behavior in children from an individual level using a social cognitive perspective. The Social Information Processing (SIP) Theory, established by renowned American psychologists Crick and Dodge (1994), is the most prominent theory in this regard as it elucidates the underlying mechanisms that influence aggressive behavior in children through individual cognitive schemas. This theory suggests that difficulties in processing social cues impede children’s ability to generate appropriate behavioral responses in their daily lives. The SIP theory not only examines the mechanism of children’s aggressive behavior from the perspective of SIP, but also underscores the significance of enhancing children’s cognitive behavioral abilities. It posits that interventions aimed at improving children’s SIP skills can effectively diminish the likelihood of children displaying aggressive behavior (Terzian et al., 2015; Wu et al., 2016). Therefore, SIP skills play a pivotal role between social skills training and children’s aggressive behavior (Spence, 2003).

Previous research has primarily focused on examining the relationship between social skills training and children’s SIP skills, as well as their aggressive behavior, which has yielded inconsistent findings across different studies (Wu et al., 2016). Gaining a comprehensive understanding of this relationship and unraveling the interconnectedness among social skills training, SIP skills, and children’s aggressive behavior is crucial for developing effective early intervention programs aimed at preventing behavioral problems in children. Furthermore, these research findings can provide empirical support for the efficacy of SIP theory in enhancing children’s behavior through targeted social skills training. Therefore, building upon the theoretical framework of SIP and utilizing data from a large-scale intervention conducted under the “Let’s Be Friends (LBF)” program with a randomized controlled experimental design, this study employs structural equation modeling (SEM) to thoroughly explore the influencing paths of social skills training on children’s cognitive concentration as the primary outcome variable while simultaneously exploring the mediating role played by SIP skills.

## 2 Literature review

Aggressive behavior is an important category of deviant conduct that is prevalent among children worldwide (Crick and Grotpeter, 1995; Zhong et al., 2014). Macgowan et al. (2002) categorized children’s aggressive behavior into physical aggression, relational aggression, social engagement, and cognitive concentration at both the peer group and individual levels. Cognitive concentration refers to the skills, attitudes, and behaviors exhibited by children during their learning process. It encompasses a range of personal-level attributes that can impact a child’s academic success in the classroom. These factors include protective elements such as eagerness to learn and perseverance in task completion, as well as risk factors like susceptibility to distractions or lack of effort. Children with low cognitive concentration primarily demonstrate poor school readiness which leads to disengagement from learning and lower academic performance (Eron, 1997; Hawkins et al., 1998; Lipsey and Derzon, 1998; Loeber, 1998). Pearson correlation coefficient tests conducted by Macgowan et al. (2002) revealed that cognitive concentration showed the highest correlation with grade point average (GPA) compared to the other three types of aggressive behavior. Existing theories and research convincingly demonstrate that early childhood aggression serves as an important indicator for predicting long-term adverse developmental trajectories (Macgowan et al., 2002). Without effective intervention, children are more likely to encounter persistent learning difficulties and behavioral problems during late elementary school years and middle school stages (Chen et al., 2017; Denham et al., 2013), which may even have implications on their lifelong behavioral patterns, especially for children facing socio-economic disadvantages.

Various models have been employed by scholars to elucidate the genesis and progression of aggressive behavior in children, among which Dodge’s (1986) SIP model has exerted a substantial influence. Initially focused on delineating internalizing disorders in children (Dodge, 1993), this model underwent revision proposed by Crick and Dodge (1994) in order to investigate externalizing disorders in children. Falling within the domain of cognitive psychology, the SIP theory posited by Crick and Dodge (1994) postulates that interpersonal behaviors exhibited by children within specific social contexts are ultimate manifestations resulting from a series of steps involving SIP utilizing internal cognitive representations for external stimuli processing such as peer provocation. Early aggressive behavior in children is frequently associated with deficiencies in their cognitive and SIP skills, with numerous studies corroborating a significant correlation between these two factors (Lengua, 2002; Pang and Tian, 2002). Deficient cognition during childhood can be likened to automatically occurring stereotypes (Dodge, 2006), which if left unaddressed promptly may become ingrained during adolescence. Each step involved in SIP under this cognitive representation possesses the potential to engender enduring aggressive behavior patterns in children.

The SIP theory not only explores the cognitive mechanisms underlying children’s social behavior but also emphasizes the significance of initiating cognitive training to rectify the behavior in children. Scholars have conducted longitudinal and experimental studies on the temporal association between children’s SIP problems and aggressive behavior, revealing that deficiencies in children’s SIP can exert an influence on subsequent aggressive behavior (Rabiner and Coie, 1989). Interventions aimed at preventing aggressive behavior leverage these temporal relationships by implementing comprehensive intervention programs to enhance children’s processing skills for social information, which has been empirically demonstrated as effective in reducing aggressive behavior (Slaby and Guerra, 1990; Hudley and Graham, 1993). Therefore, early provision of efficacious social skills training programs for children is pivotal for enhancing their processing skills for social information and mitigating aggressive behavior in this population (Dodge and Godwin, 2013).

Previous research has not conducted a statistical analysis on the mediating effect of enhancing children’s SIP skills through social skills training on their aggressive behavior (De Castro et al., 2005; Pakaslahti, 2000). However, Crick and Dodge (1994) explicitly emphasized that the mediation model is most suitable for understanding the processing process of a single stimulus, such as an intervention, although it may be challenging to examine each stage in detail. If the existing theory accurately describes the corresponding mediating mechanism, then the mediation model can be employed to test this process (Pössel and Hautzinger, 2006). In this case, there is no need for a detailed analysis of each stage of a single stimulus processing process; rather, it is sufficient to understand the output at each stage, such as children’s level of SIP skills and cognitive concentration. This article utilizes SIP theory as a robust theoretical framework to enhance children’s cognitive concentration by improving their SIP skills. The social skills training program developed based on this theory is widely recognized as an effective intervention strategy. Therefore, SIP skills play a crucial mediating role between social skills training and children’s cognitive concentration. The proposed mediating effect model can be constructed and its influencing mechanism verified by examining relationships among social skills training, different stages of SIP skills, and results of children’s cognitive concentration.

## 3 Theoretical framework and hypotheses

The core theory relied upon in this study is the SIP theory proposed by Crick and Dodge (1994). The SIP theory aims to elucidate the development of children’s aggressive behavior from a SIP perspective. This theory conceptualizes the cognitive process of children, encompassing six sequential steps: encoding of cues, interpretation of cues, clarification of goals, response access or construction, response decision and behavior enactment. The Skill-Level Activity (SLA) scale developed by Dodge (1980), serves as the measurement tool for assessing children’s SIP skills. However, due to challenges associated with evaluating “interpretation of cues” and “response access or construction” through questionnaires (Zhong et al., 2014), SLA primarily assesses children’s SIP skills across four stages: encoding, hostile attribution, goal formulation, and response decision. Based on empirical data validation, the SIP theory posits that cognitive deficits at each of these four stages are closely linked to children’s aggressive behavior: deficits in “encoding” lead to heightened sensitivity towards hostile cues while disregarding non-hostile cues (Dodge et al., 1990); deficits in “hostile attribution” increase the likelihood of making hostile attributions (Dodge et al., 1990); deficits in “goal formulation” result in more positive evaluations of potential outcomes related to aggressive behavior (Crick and Ladd, 1990); finally, deficits in “response decision” predispose individuals towards implementing aggressive behavior. In summary, each deficit at every stage amplifies the probability of engaging in aggressive behavior among children (Crick and Dodge, 1994; De Castro et al., 2005; Yoon et al., 1999). Therefore, the SIP theory presents compelling evidence for implementing cognitive-level interventions to enhance children’s SIP skills, thereby reducing the likelihood of aggressive behavior in children.

Social skills training is an approach that frequently employs operant conditioning techniques to teach individuals particular social behaviors by demonstrating and offering feedback, while also encouraging active participation through positive reinforcement (Spence, 2003). Let’s Be Friends (LBF) is a social skills training program for children, adapted from the “Making Choices: Social Problem-Solving for Children (MC)” program in the United States. The primary objective of the MC is to enhance children’s social and emotional skills, with a specific focus on their social cognition and based on the SIP theory. Each module in the MC curriculum corresponds to a particular step in SIP or an emotional regulation skill. Previous research has demonstrated the effective promotion of social competence through the MC program.

The preliminary research findings of “LBF (Shaanxi)” have demonstrated that interventions targeting children’s social skills can positively impact their cognitive concentration and significantly reduce aggressive behavior. Moreover, significant differences were observed between the experimental group and control group in terms of SIP skills (Guo et al., 2020). Therefore, it is essential to establish an intermediary model using correlation analysis and regression models to examine whether any observed differences in SIP skills are associated with the intervention, and whether these differences are linked to variations in children’s aggressive behavior (primarily cognitive concentration in this study) through SEM. In addition, we incorporated random controls for potential maturation effects to help ensure that any observed relationships are not solely attributable to developmental changes over time. The mediation model is crucial for comprehending influence mechanisms (Hayes and Preacher, 2014), so the purpose of this study is to develop hypotheses for more complex system relationships and processes based on the preliminary research findings of the “LBF (Shaanxi) program. Furthermore, we aim to uncover the underlying influence mechanisms (Cohen, 2003; Kenny, 1998) in order to provide a basis for effective intervention strategies that can effectively reduce children’s aggressive behavior.

**Figure 1:**
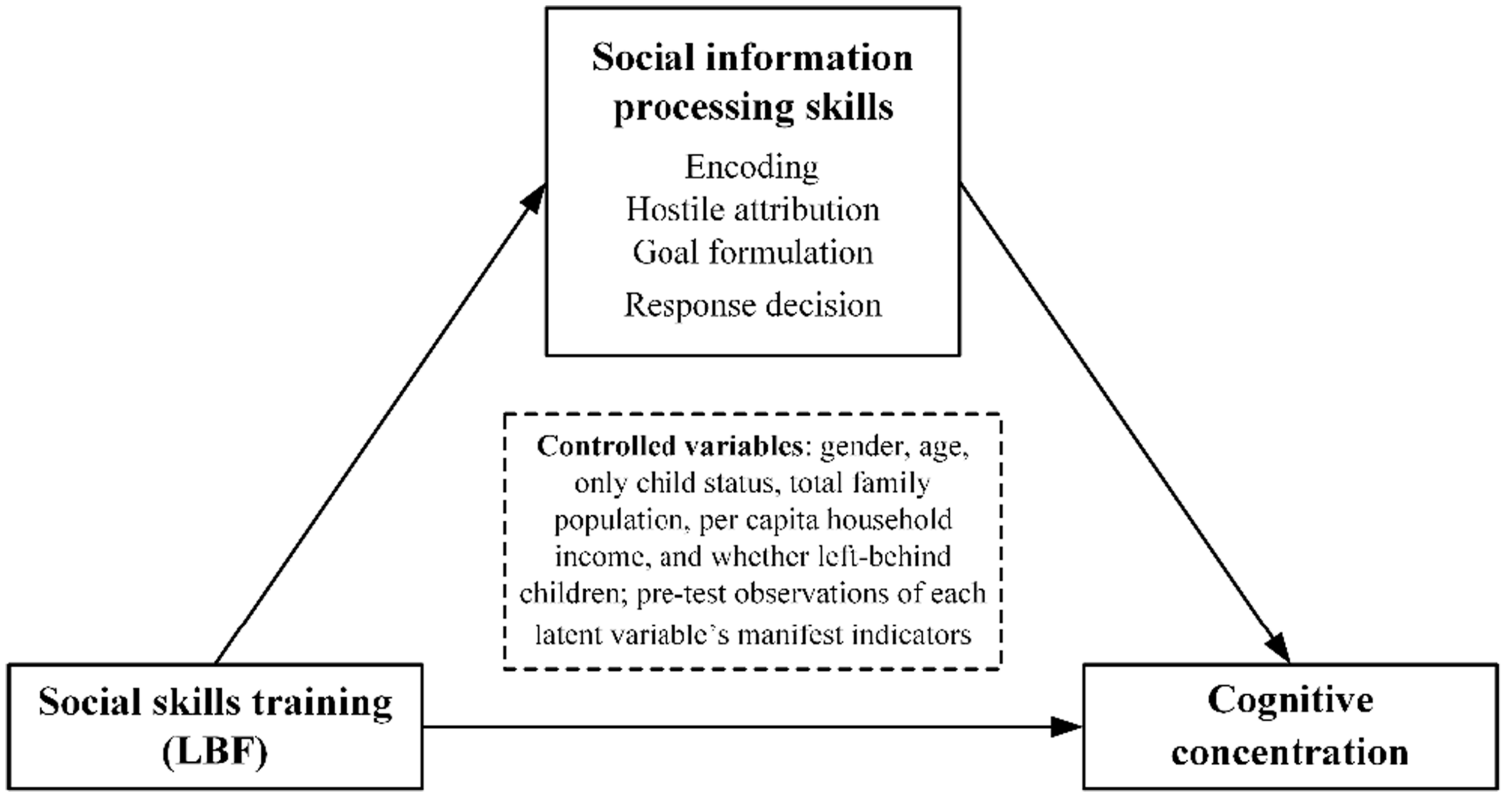
Theoretical framework.

Based on the above analysis, the theoretical framework of this paper is illustrated in Figure 1.

In addition, there are two points that require further elucidation in the theoretical framework. Firstly, most SIP literature defines the various steps of SIP as discrete processes individuals undergo when encountering social stimuli, without assuming interdependence between these steps (Lansford et al., 2006). Furthermore, previous research has demonstrated that children with concurrent cognitive deficits across multiple steps of SIP exhibit higher scores on externalizing behavior problems compared to children who only experience difficulties in one step (Lansford et al., 2006). Therefore, this study also treats each step of SIP as an independent process and analyzes them separately as mediating variables. Secondly, previous research has indicated a higher prevalence of problems with SIP and aggressive behavior among boys compared to girls (Lansford et al., 2006; Macgowan et al., 2002). Hence, it is imperative to conduct gender-based grouping to explore whether significant gender differences exist in the influencing paths where a child’s social skills training affects their SIP skills and subsequently influences their cognitive concentration level.

Based on the aforementioned analysis, we propose the following hypotheses:

H_1-1_: A positive correlation exists between social skills training and encoding.

H_1-2_: A negative correlation exists between social skills training and hostile attribution.

H_1-3_: A positive correlation exists between social skills training and goal formulation.

H_1-4_: A positive correlation exists between social skills training and response decision.

H_2_: Social skills training has a direct positive relationship with children’s cognitive concentration.

H_2-1_: Social skills training positively influences children’s cognitive concentration through encoding.

H_2-2_: Social skills training positively influences children’s cognitive concentration through hostile attribution.

H_2-3_: Social skills training positively influences children’s cognitive concentration through goal formulation.

H_2-4_: Social skills training positively influences children’s cognitive concentration through response decision.

H_3_: Gender differences exist in the mediating effects of children’s SIP skills on the relationship between social skills training and cognitive concentration.

## 4 Research design

### 4.1 Data

The data for this study was obtained from the “LBF (Shaanxi)” intervention program, conducted by a research team from X University in China and W University in the USA, between September 2018 and January 2019 in J County, Shaanxi Province. LBF is the Chinese adaptation of MC and comprises 8 modules and encompasses 31 lessons. It has undergone testing in Tianjin and Shaanxi provinces (Guo et al., 2020; Wu et al., 2016). LBF (Shaanxi) intervention comprised a total of 14 weekly sessions conducted from September to December in 2018. Building upon the insights gained from the Tianjin pilot test, the Shaanxi program condensed the initial set of 31 lessons into these 14 sessions, each lasting approximately 60 min. All 31 lessons were effectively covered during this intervention. Considering the size of certain schools, the intervention was delivered in 15 groups, with two to three trainers assigned to each group. In total, there were 29 trainers involved across all participating schools. These trainers received university credit for their active engagement in the program and also received modest compensation for any incurred expenses such as travel costs. To ensure that the student-to-teacher ratio did not exceed 10, when the number of students in a third-grade class exceeds 30, we split the class into two separate classes. Each class was staffed with one trainer and two teaching assistants during every session. Therefore, the teaching assistants could provide the necessary support when needed. The research team also included supervisors, who monitored the program and resolved unexpected problems. A detailed treatment manual, rehearsal before each session, and lesson process reports were created to maintain fidelity (Guo et al., 2020).

LBF (Shaanxi) program adhered to a rigorous ethical protocol. It received approval from local educational authorities. Before the intervention commenced, all research and intervention staff underwent comprehensive ethics training. After a detailed explanation of the study’s objectives, procedures, potential risks, and benefits, informed consent was obtained from all child participants’ legal guardians, who signed confidentiality and service agreements. All personal information was kept confidential and anonymized to protect participant privacy. These procedures ensured full compliance with institutional and local regulations, safeguarding participant rights and ensuring data integrity throughout the study.

Guided by SIP theory and intervention research theory, the program aimed to enhance children’s SIP skills and social behaviors (including cognitive concentration that notably impact academic performance) through a four-month behavioral intervention targeting children aged 8–10 years old. The “LBF (Shaanxi)” program employed a Randomized Controlled Trial (RCT) design with fully blocked randomization to select the experimental group (14 schools with 350 primary school students), which was matched with control group schools (14 schools with 350 primary school students) using Mahalanobis Metric Distance based on school-level data to ensure high internal validity of the intervention effect evaluation while avoiding spill-over effects. The study had sufficient power to detect small to moderate effect sizes. Consequently, there were ultimately 13 experimental group schools (355 third-grade students) and 14 control group schools (341 third-grade students). The gender ratio (male-to-female proportion) of the participants is 48.3% boys and 51.7% girls.^1^

### 4.2 Measures

The dependent variable is children’s cognitive concentration, which is measured by the Carolina Child Checklist (CCC) developed by the Carolina Children’s Initiative. This initiative project aims to prevent aggressive behavior in children. The CCC conceptual framework emphasizes the important role of individual and peer factors in the risk process leading to childhood aggression. Completed primarily by teachers or social workers, the checklist evaluates the frequency of discrete behaviors and attributes exhibited by a child rather than providing an overall assessment of specific attributes possessed by a child as a whole. Furthermore, it enables capturing gender-related differences in behavioral patterns (Macgowan et al., 2002).

Cognitive concentration, as the sub-dimension with the highest weight among the 10 sub-dimensions of CCC, represents individual-level classroom learning performance in children. It is primarily measured through 12 items including ability to work independently, absent-mindedness (reversed), did not study hard (reversed), complete missions successfully, easily distracted (reversed), the initiative to learn to improve the ability, willing to study, ability to focus on a task, concentration, study hard, attention focusing, and independent. Teachers observe children’s behavior over the past month and rate each item on a scale ranging from 0 (never) to 5 (always). The measurement of CCC serves two purposes: firstly, it assesses behavioral domains corresponding to risk factors and protective factors associated with childhood aggressive behavior; secondly, it evaluates sensitivity towards short-term intervention effects. Therefore, this study adopts CCC as a measurement tool to evaluate both children’s cognitive concentration levels and reflect the effectiveness of social skills training program (LBF).

The mediating variable is children’s SIP skills, which is primarily measured by the “Skill-Level Activity (SLA) scale” developed by Dodge (1980). The SLA assesses how children interpret and respond to specific interpersonal vignettes. Through pictures and stories, children envision themselves in five hypothetical scenarios: watching a “dodgeball game,” attending a “math class,” wearing “new pants,” eating “lunch,” and experiencing the loss of a “new magazine.” A grading rubric evaluates their competencies across four SIP skill areas—encoding (α = 0.78), hostile attribution (α = 0.52), goal formulation (α = 0.76), and response decision (α = 0.80)—with responses from each scenario influencing their scores. “Encoding” is assessed based on the number of cues children interpret in each scenario, with scoring ranges varying by story: 0–4 for the “new magazine,” 0–5 for the “math class” and “lunch,” and 0–6 for the “dodgeball” and “new pants.” “Hostile attribution” evaluates whether children perceive hostility in the scenarios, with 0 indicating an absence of hostile attribution and 1 indicating its presence. “Goal formulation” examines the nature of the goals children set, with 0 for aggressive intentions and 1 for non-aggressive ones. Lastly, “Response decision” measures the aggressiveness of children’s responses and actions, with scores of 0 for aggressive and 1 for non-aggressive behaviors.

The independent variable in this study is whether children received social skills training from the “LBF (Shaanxi)” program. Children who received the intervention in the experimental group are coded as 1, while those who did not receive any intervention in the control group are coded as 0. The controlled variables primarily include children’s gender, age, only child status, total family population, per capita household income, and whether they are left-behind children. Additionally, pre-test observations of each latent variable’s manifest indicators are included as controlled variables in the model.

The distribution of the sample and descriptive statistics for each variable are presented in Table 1. After excluding students who need special assistance, the final sample consisted of 681 participants, with 343 children in the experimental group (accounting for 50.4% of the total) and 338 children in the control group (accounting for 49.6% of the total). Among them, there were 329 boys (48.3% of the total) and 352 girls (51.7% of the total), with an average age of 8.689 years old.

**Table 1:** Descriptive statistics of variables (*N* = 681).

Latent variables	Observational variables	Mean	SD
Cognitive concentration (post-test)	Y1 ability to work independently (0–5)	3.144	1.232
	Y2 absent-mindedness (reversed) (0–5)	3.507	1.050
	Y3 did not study hard (reversed) (0–5)	3.687	1.199
	Y4 complete missions successfully (0–5)	3.167	1.192
	Y5 easily distracted (reversed) (0–5)	3.379	1.197
	Y6 the initiative to learn to improve the ability (0–5)	3.188	1.260
	Y7 willing to study (0–5)	3.483	1.279
	Y8 ability to focus on a task (0–5)	2.919	1.269
	Y9 concentration (0–5)	3.060	1.243
	Y10 study hard (0–5)	3.264	1.202
	Y11 attention focusing (0–5)	3.109	1.176
	Y12 independent (0–5)	2.912	1.262
SIP: Encoding (post-test)	Y13 Dodgeball (0–6)	3.825	1.692
	Y14 Math class (0–5)	3.065	1.403
	Y15 New pants (0–6)	3.587	1.672
	Y16 Lunch (0–5)	3.781	1.538
	Y17 New magazine (0–4)	2.242	1.040
SIP: Hostile attribution (post-test)	Y18 Dodgeball (0–1)	0.474	0.500
	Y19 Math class (0–1)	0.621	0.485
	Y20 New pants (0–1)	0.317	0.466
	Y21 Lunch (0–1)	0.605	0.489
	Y22 New magazine (0–1)	0.452	0.498
SIP: Goal formulation (post-test)	Y23 Dodgeball (0–1)	0.746	0.436
	Y24 Math class (0–1)	0.862	0.345
	Y25 New pants (0–1)	0.928	0.259
	Y26 Lunch (0–1)	0.862	0.345
	Y27 New magazine (0–1)	0.361	0.481
SIP: Response decision (post-test)	Y28 Dodgeball (0–1)	0.897	0.304
	Y29 Math class (0–1)	0.932	0.251
	Y30 New pants (0–1)	0.896	0.306
	Y31 Lunch (0–1)	0.474	0.500
	Y32 New magazine (0–1)	0.869	0.337
Cognitive concentration (pre-test)	X1 ability to work independently (0–5)	3.007	1.282
	X2 absent-mindedness (reversed) (0–5)	3.194	1.177
	X3 did not study hard (reversed) (0–5)	3.464	1.252
	X4 complete missions successfully (0–5)	2.979	1.179
	X5 easily distracted (reversed) (0–5)	3.041	1.265
	X6 the initiative to learn to improve the ability (0–5)	2.938	1.319
	X7 willing to study (0–5)	3.292	1.334
	X8 ability to focus on a task (0–5)	2.606	1.257
	X9 concentration (0–5)	2.903	1.173
	X10 study hard (0–5)	3.050	1.248
	X11 attention focusing (0–5)	2.831	1.169
	X12 independent (0–5)	2.762	1.145
SIP: Encoding (pre-test)	X13 Dodgeball (0–6)	1.981	1.311
	X14 Math class (0–5)	1.392	0.799
	X15 New pants (0–6)	1.802	1.206
	X16 Lunch (0–5)	1.786	1.710
	X17 New magazine (0–4)	1.329	0.726
SIP: Hostile attribution (pre-test)	X18 Dodgeball (0–1)	0.476	0.500
	X19 Math class (0–1)	0.740	0.439
	X20 New pants (0–1)	0.408	0.492
	X21 Lunch (0–1)	0.661	0.474
	X22 New magazine (0–1)	0.567	0.496
SIP: Goal formulation (pre-test)	X23 Dodgeball (0–1)	0.577	0.494
	X24 Math class (0–1)	0.800	0.400
	X25 New pants (0–1)	0.890	0.313
	X26 Lunch (0–1)	0.712	0.453
	X27 New magazine (0–1)	0.269	0.444
SIP: Response decision (pre-test)	X28 Dodgeball (0–1)	0.761	0.427
	X29 Math class (0–1)	0.888	0.315
	X30 New pants (0–1)	0.827	0.379
	X31 Lunch (0–1)	0.404	0.491
	X32 New magazine (0–1)	0.784	0.412
Independent variable	X33 LBF intervention (1 = LBF group, 0 = control group)	0.504	0.500
Control variables	X34 Gender (1 = boy, 0 = girl)	0.483	0.500
	X35 age (age at pre-test)	8.689	0.471
	X36 only child status (1 = only child, 0 = not only child)	0.289	0.454
	X37 total family population (1–10)	5.091	1.215
	X38 per capita household income (1–9)	2.570	2.190
	X39 whether left-behind children (1 = left-behind, 0 = not left-behind)	0.320	0.467

### 4.3 Method

Given the need to simultaneously estimate the relationships between measurement indicators and latent variables, as well as the relationships between latent variables, it is imperative to ascertain the paths and mechanisms through which intervention impacts children’s cognitive concentration via their SIP skills. Therefore, a structural equation model (SEM) is employed. The analysis of mediation effects primarily draws upon Baron and Kenny’s (1986) proposed process for testing mediation models. Mediation effects can only exist when the independent variable significantly influences the dependent variable, with such effects diminishing the impact of the independent variable on the dependent variable. If, at this juncture, there remains a significant influence of the independent variable on the dependent variable, it indicates partial mediation effects. Conversely, if mediation effects render insignificant any influence of the independent variable on the dependent variable, it signifies full mediation effects. Statistical analysis in this study was conducted using STATA 17.0.

**Figure 2:**
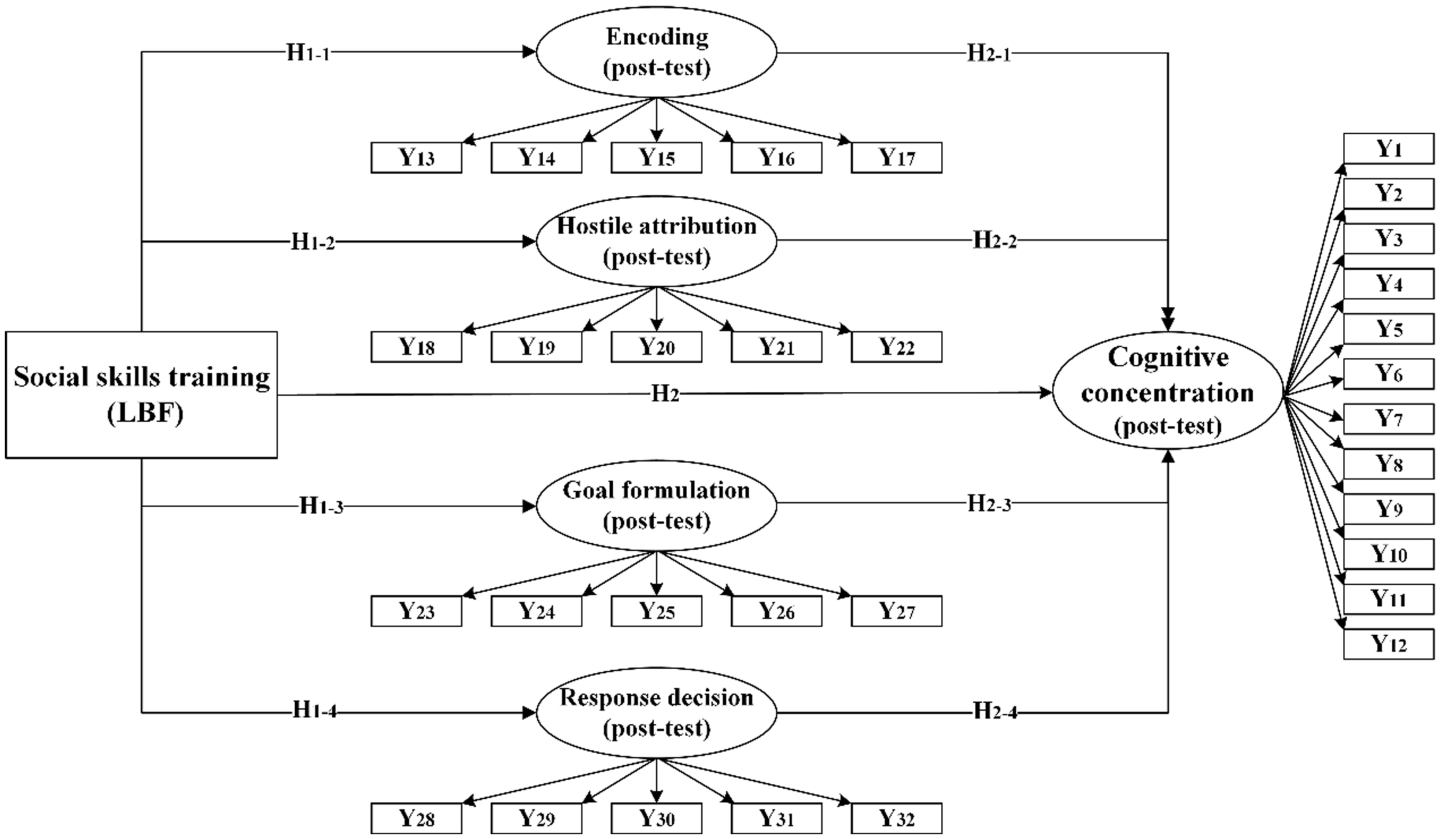
Structural equation model.

According to the theoretical framework (see Figure 1) and research hypotheses, a structural equation model is established for analysis (see Figure 2). The mapping between latent variables and measurement indicators, as well as the control variables, are illustrated in Table 1. Specifically, firstly, social skills training exerts a direct impact on post-test values of encoding, hostile attribution, goal formulation, and response decision within SIP. Previous studies have demonstrated that children’s SIP skills are notably influenced by exogenous factors. Therefore, social skills training is considered an exogenous variable. Secondly, social skills training has both direct and indirect effects on children’s cognitive concentration. The indirect effect occurs through its influence on children’s SIP skills (including encoding, hostile attribution, goal formulation, and response decision). Thirdly, gender differences are examined by categorizing boys and girls into groups to investigate how social skills training affects children’s cognitive concentration through different stages of their SIP skills.

## 5 Results

### 5.1 Goodness of fit for structural equation models

The fit indices of the structural equation model are crucial for elucidating the relationship between the measurement model and latent variables. Typically, *χ^2^* (Chi-square), *χ^2^/df* (Chi-square/Degrees of Freedom), RMSEA (Root Mean Square Error of Approximation), SRMR (Standardized Root Mean Square Residual), CFI (Comparative Fit Index), TLI (Tucker–Lewis Index), and CD (Coefficient of Determination) serve as primary indices to assess the goodness-of-fit. Smaller values of *χ^2^* and *χ^2^/df* are indicative of a better fit; generally less than 5 is considered favorable (Li and Qiu, 2016). CFI, TLI, and CD are evaluation metrics provided by STATA software to gauge the adequacy of structural equation models. The closer their values approach 1, the more optimal the model fits; typically greater than 0.8 is deemed satisfactory. Lower values of RMSEA and SRMR indicate a superior fit; commonly accepted thresholds for acceptable models are below 0.08 while below 0.05 is considered excellent (Kline, 2015; Markus, 2012). The specific fit indices for both total sample and sub-sample can be found in Table 2. In our hypothesized model, *χ^2^/df* = 2.363 < 5, RMSEA = 0.045 < 0.05, SRMR = 0.048 < 0.05, CFI = 0.851 > 0.8, TLI = 0.841 > 0.8. The overall fit indices from boys’ and girls’ samples suggest that our hypothesized model adequately captures the underlying structural relationships within the sample data structure.

**Table 2:** Structural model fitting indicators.

Fitting indicators	All samples	Boy samples	Girl samples
*χ^2^*	5460.378	4190.508	3959.927
*df*	2,311	2,257	2,257
*χ^2^/df*	2.363	1.857	1.755
RMSEA	0.045	0.051	0.046
P (RMSEA < 0.05)	1.000	0.223	0.994
CFI	0.851	0.817	0.834
TLI	0.841	0.805	0.824
SRMR	0.048	0.059	0.057
CD	1.000	1.000	1.000
Sample size	681	329	352

CD, Coefficient of determination.

The relationship between the observed variables and latent variables is presented in Table 3. The results indicate that the factor loadings of the observed variables are statistically significant, with most indicators exhibiting factor loadings above 0.5. This suggests a high level of validity for the measured variables, indicating their effectiveness in assessing the latent constructs. It is noteworthy that within the measurement model, standardized factor loadings for specific story contexts such as “dodgeball” in hostile attribution, “math class” and “new magazine” in goal formulation, and “lunch” in response decision fall below 0.4. This implies that these particular story contexts may not adequately capture variations across different stages of SIP when assessed individually. Further research should be conducted to address this limitation concerning SIP measurement at distinct stages.

**Table 3:** The fit of the measurement model (*N* = 681).

Latent variables	Observational variables	Standardized factor loading	The square of the bivariate relationship coefficient
Cognitive concentration (post-test)	Y1 ability to work independently	0.788	0.621
	Y2 absent-mindedness (reversed)	0.610	0.372
	Y3 did not study hard (reversed)	0.673	0.453
	Y4 complete missions successfully	0.775	0.601
	Y5 easily distracted (reversed)	0.516	0.266
	Y6 the initiative to learn to improve the ability	0.866	0.750
	Y7 willing to study	0.882	0.778
	Y8 ability to focus on a task	0.784	0.615
	Y9 concentration	0.817	0.668
	Y10 study hard	0.909	0.825
	Y11 attention focusing	0.854	0.730
	Y12 independent	0.534	0.286
SIP: Encoding (post-test)	Y13 Dodgeball	0.860	0.739
	Y14 Math class	0.839	
	Y15 New pants	0.846	0.715
	Y16 Lunch	0.625	0.390
	Y17 New magazine	0.687	0.472
SIP: Hostile attribution (post-test)	Y18 Dodgeball	0.399	0.159
	Y19 Math class	0.473	0.224
	Y20 New pants	0.478	0.228
	Y21 Lunch	0.510	0.260
	Y22 New magazine	0.600	0.359
SIP: Goal formulation (post-test)	Y23 Dodgeball	0.437	0.191
	Y24 Math class	0.343	0.118
	Y25 New pants	0.685	0.469
	Y26 Lunch	0.444	0.197
	Y27 New magazine	0.085	0.006
SIP: Response decision (post-test)	Y28 Dodgeball	0.464	0.216
	Y29 Math class	0.708	0.501
	Y30 New pants	0.700	0.489
	Y31 Lunch	0.195	0.038
	Y32 New magazine	0.630	0.397
Cognitive concentration (pre-test)	X1 ability to work independently	0.800	0.640
	X2 absent-mindedness (reversed)	0.663	0.439
	X3 did not study hard (reversed)	0.695	0.483
	X4 complete missions successfully	0.839	0.704
	X5 easily distracted (reversed)	0.608	0.369
	X6 the initiative to learn to improve the ability	0.855	0.732
	X7 willing to study	0.860	0.740
	X8 ability to focus on a task	0.730	0.533
	X9 concentration	0.839	0.704
	X10 study hard	0.905	0.819
	X11 attention focusing	0.860	0.739
	X12 independent	0.654	0.427
SIP: Encoding (pre-test)	X13 Dodgeball	0.709	0.503
	X14 Math class	0.601	0.362
	X15 New pants	0.764	0.584
	X16 Lunch	0.581	0.338
	X17 New magazine	0.539	0.290
SIP: Hostile attribution (pre-test)	X18 Dodgeball	0.385	0.148
	X19 Math class	0.408	0.166
	X20 New pants	0.453	0.205
	X21 Lunch	0.495	0.245
	X22 New magazine	0.637	0.406
SIP: Goal formulation (pre-test)	X23 Dodgeball	0.253	0.064
	X24 Math class	0.297	0.088
	X25 New pants	0.532	0.283
	X26 Lunch	0.327	0.107
	X27 New magazine	0.080	0.006
SIP: Response decision (pre-test)	X28 Dodgeball	0.358	0.129
	X29 Math class	0.471	0.221
	X30 New pants	0.578	0.334
	X31 Lunch	0.121	0.015
	X32 New magazine	0.499	0.249

(1) The first indicator of the latent variable is used as the reference scale. (2) Two-tailed test significance: ^+^*p* < 0.1, *p < 0.05, **p < 0.01, ***p < 0.001.

### 5.2 Influencing paths of the impact of social skills training on children’s cognitive concentration

**Figure 3:**
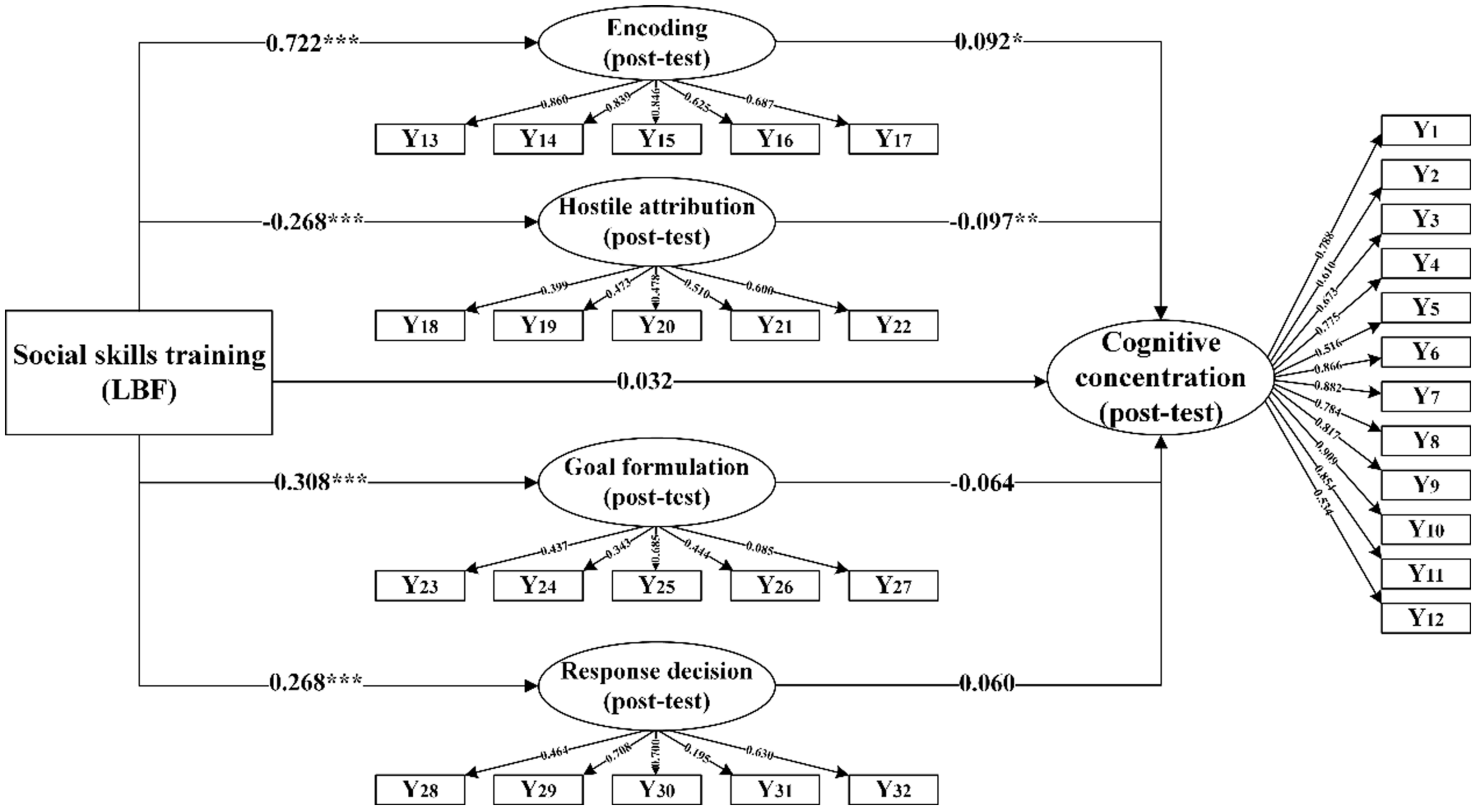
Path diagram of the effects of social skills training (LBF) on children’s cognitive concentration.

The path diagram and model testing results for the impact of social skills training on children’s cognitive concentration are shown in Figure 3 and Table 4.

**Table 4:** The standardized path coefficient of social skills training on children’s cognitive concentration (*N* = 681).

Variables	SIP: Encoding (post-test)	SIP: Hostile attribution (post-test)	SIP: Goal formulation (post-test)	SIP: Response decision (post-test)	Cognitive concentration (post-test)
LBF intervention	0.722^***^ (0.019)	−0.268^***^ (0.045)	0.308^***^ (0.054)	0.268^***^ (0.041)	0.032 (0.043)
SIP: Encoding (post-test)					0.092^*^ (0.043)
SIP: Hostile attribution (post-test)					−0.097^**^ (0.038)
SIP: Goal formulation (post-test)					−0.064 (0.101)
SIP: Response decision (post-test)					0.060 (0.097)
Pre-test of each variable	0.245^***^ (0.031)	0.507^***^ (0.052)	0.802^***^ (0.047)	0.776^***^ (0.034)	0.729^***^ (0.023)
Structural equation explanation percentages (%)	57.07	35.25	68.35	65.21	56.83

(1) The independent variables in the row, while the dependent variables in the column. (2) Standard error in parentheses. (3) Two-tailed test significance: ^+^*p* < 0.1, * *p* < 0.05, ** *p* < 0.01, *** *p* < 0.001.

The model in this study accounts for 57.07% of the variance in encoding (post-test), 35.25% of the variance in hostile attribution (post-test), 68.35% of the variance in goal formulation (post-test), 65.21% of the variance in response decision (post-test), and 56.83% of the variance in cognitive concentration (post-test). The findings suggest that social skills training influences children’s cognitive concentration by impacting their skills to process social information.

#### 5.2.1 The impact of social skills training on children’s SIP skills

In the analysis that controlled for pre-test SIP and other variables, it was observed that LBF intervention had a significant impact on all steps of SIP. Specifically, the standardized path coefficient between the social skills training and encoding was found to be 0.722 (*p* < 0.001), thereby verifying hypothesis H_1-1_. The standardized path coefficient between LBF intervention and hostile attribution was −0.268 (*p* < 0.001), supporting hypothesis H_1-2_. Additionally, hypothesis H_1-3_ was validated as well with a standardized path coefficient of 0.308 (*p* < 0.001), indicating a positive correlation between the social skills training and goal formulation. While hypothesis H_1-4_ received confirmation with a standardized path coefficient of 0.268 (*p* < 0.001), suggesting a positive correlation between LBF intervention and response decision. Fundamentally, these findings suggest that interventions targeting children’s SIP skills can effectively enhance their abilities across all four steps of SIP.

#### 5.2.2 The impact of social skills training on children’s cognitive concentration

Regarding direct effects, after controlling for pre-test SIP, pre-test cognitive concentration, and other variables, the standardized path coefficient of LBF on children’s cognitive concentration is 0.032 and lacks statistical significance. This suggests that there is no direct impact of LBF intervention on children’s cognitive concentration; thus, hypothesis H_2_ cannot be supported.

In terms of indirect effects, after controlling for pre-test SIP, pre-test cognitive concentration, and other variables, the standardized path coefficient of LBF intervention on encoding was 0.722 (*p* < 0.001), indicating a significant positive relationship. Additionally, the standardized path coefficient of encoding on cognitive concentration was 0.092 (*p* < 0.05), suggesting a significant association. Thus, it can be concluded that encoding mediate the relationship between LBF intervention and children’s cognitive concentration, supporting hypothesis H_2-1_. Similarly, the standardized path coefficient of LBF intervention on hostile attribution was −0.268 (*p* < 0.001), demonstrating a significant negative impact; whereas the standardized path coefficient of hostile attribution on cognitive concentration was −0.097 (*p* < 0.01), indicating another significant link between these variables. Consequently, it can be inferred that hostile attribution mediates the relationship between LBF intervention and children’s cognitive concentration in line with hypothesis H_2-2_. Despite observing a significant positive effect of LBF intervention on goal formulation and response decision, neither goal formulation nor response decision had a statistically significant influence on children’s cognitive concentration; thus hypotheses H_2-3_ and H_2-4_ were not supported.

In conclusion, LBF intervention does not directly impact children’s cognitive concentration; rather, it exerts its influence through the mediating role of encoding and hostile attribution in SIP skills. SIP skills play a fully mediating role between social skills training and children’s cognitive concentration.

#### 5.2.3 Standardized total effects on children’s cognitive concentration

The standardized total effects of factors on children’s cognitive concentration are further reported in Table 5. Consistent with the aforementioned analysis results, the core finding indicates that LBF intervention has a significant impact on enhancing children’s cognitive concentration, which is fully mediated by their SIP skills.

**Table 5:** Standardized total effect of each factor on children’s cognitive concentration (*N* = 681).

Variables	SIP: Encoding (post-test)	SIP: Hostile attribution (post-test)	SIP: Goal formulation (post-test)	SIP: Response decision (post-test)	Cognitive concentration (post-test)
LBF intervention	0.722^***^ (0.019)	−0.268^***^ (0.045)	0.308^***^ (0.055)	0.268^***^ (0.041)	0.120^***^ (0.029)
SIP: Encoding (post-test)					0.092^*^ (0.043)
SIP: Hostile attribution (post-test)					−0.097^*^ (0.039)
SIP: Goal formulation (post-test)					−0.064 (0.119)
SIP: Response decision (post-test)					0.060 (0.115)

(1) The independent variables in the row, while the dependent variables in the column. (2) Standard error in parentheses. (3) Two-tailed test significance: ^+^*p* < 0.1, *p < 0.05, **p < 0.01, ***p < 0.001.

### 5.3 Gender differences of social skills training’s influences on children’s cognitive concentration through SIP skills

Based on the findings presented in Table 6, it can be inferred that LBF intervention significantly influences the SIP skills of both genders. Specifically, for girls, the standardized path coefficients of LBF intervention on encoding, hostile attribution, goal formulation, and response decision are 0.720 (*p* < 0.001), −0.323 (*p* < 0.001), 0.278 (*p* < 0.001), and 0.204 (*p* < 0.01) respectively. For boys, the standardized path coefficients of LBF intervention on encoding, hostile attribution, goal formulation, and response decision are 0.722 (*p* < 0.001), −0.210 (*p* < 0.01), 0.358 (*p* < 0.001), and 0.357 (*p* < 0.001). These results indicate a stronger impact of LBF intervention on boys’ SIP skills compared to girls.

**Table 6:** Gender differences in cognitive concentration path coefficients of children affected by social skills training (LBF).

Variables	SIP: Encoding (post-test)		SIP: Hostile attribution (post-test)		SIP: Goal formulation (post-test)		SIP: Response decision (post-test)		Cognitive concentration (post-test)		
	Girls	Boys	Girls	Boys	Girls	Boys	Girls	Boys	Girls	Boys	Differences
LBF intervention	0.720^***^ (0.027)	0.722^***^ (0.028)	−0.323^***^ (0.060)	−0.210^**^ (0.066)	0.278^***^ (0.076)	0.358^***^ (0.086)	0.204^**^ (0.063)	0.357^***^ (0.061)	0.021 (0.063)	0.025 (0.068)	−0.004 (0.093)
SIP: Encoding (post-test)									0.113^+^ (0.062)	0.104 (0.064)	0.009 (0.089)
SIP: Hostile attribution (post-test)									−0.078 (0.056)	−0.128^*^ (0.058)	0.050 (0.081)
SIP: Goal formulation (post-test)									0.009 (0.098)	−0.553^+^ (0.287)	0.544^+^ (0.303)
SIP: Response decision (post-test)									0.014 (0.097)	0.529^+^ (0.289)	−0.515^+^ (0.305)
Pre-test of each variable	0.249^***^ (0.044)	0.243^***^ (0.046)	0.448^***^ (0.073)	0.569^***^ (0.074)	0.759^***^ (0.069)	0.954^***^ (0.058)	0.671^***^ (0.056)	0.876^***^ (0.045)	0.672^***^ (0.034)	0.759^***^ (0.037)	−0.087^+^ (0.050)
Structural equation explanation percentages (%)	55.6	58.7	33.1	38.5	65.9	90.9	54.2	79.5	49.9	62.9	
Sample size	352	329	352	329	352	329	352	329	352	329	

(1) The independent variables in the row, while the dependent variables in the column. (2) Standard error in parentheses. (3) Two-tailed test significance: ^+^*p* < 0.1, *p < 0.05, **p < 0.01, ***p < 0.001.

The standardized path coefficients of LBF intervention on the cognitive concentration of girls and boys were 0.021 and 0.025, respectively, and both were not statistically significant, indicating that LBF intervention does not have a direct impact on the cognitive concentration of girls and boys. Regarding the mediating effect, the standardized path coefficient of encoding on the influence of cognitive concentration in girls was 0.113 (*p* < 0.1), suggesting that LBF intervention has a positive correlation with cognitive concentration in girls through encoding. In contrast, hostile attribution by boys had a standardized path coefficient of −0.128 (*p* < 0.05), indicating that LBF intervention has a positive correlation with cognitive concentration in boys by reducing hostile attribution. Furthermore, response decision by boys had a standardized path coefficient of 0.529 (*p* < 0.1), suggesting that LBF intervention has a positive correlation with cognitive concentration in boys through response decision. Contrary to our hypothesis, the standardized path coefficient for goal formulation’s influence on cognitive concentration in boys was −0.553 (*p* < 0.1).This result may be attributed to low factor loadings for math class and new magazine factors mentioned earlier when measuring goal formulation. It is necessary to further improve measurement tools in future research to obtain more accurate effects.

In conclusion, the results reveal significant gender disparities in the influencing path of LBF intervention impacting cognitive concentration through SIP skills. The hypothesis H_3_ is verified.

## 6 Discussion and conclusion

The level of cognitive concentration reflects children’s classroom learning skills, attitudes, and behaviors, which are crucial factors in ensuring improved academic achievement for children. Therefore, it is imperative to enhance children’s cognitive concentration. This study employed a randomized controlled experimental data analysis to examine the influencing paths of social skills training (LBF) on children’s cognitive concentration and tested the mediating mechanism of SIP skills. Pre-tests were controlled on all variables to ensure a more objective and accurate influence mechanism. Ultimately, the research findings confirm that a social skills training program focused on enhancing children’s SIP skills represents an effective strategy for augmenting their cognitive concentration.

1. Social skills training (i.e., LBF in this study) significantly enhances children’s SIP skills and cognitive concentration. It exerts a significant impact on various steps of children’s SIP skills, including encoding, hostile attribution, goal formulation, and response decision. Moreover, the overall effect of social skills training on children’s cognitive concentration is statistically significant at a significance level of 0.001, indicating its substantial effectiveness in improving both their SIP skills and cognitive concentration. These findings validate previous research conducted under the “LBF (Shaanxi)” program. Furthermore, this research elucidates the underlying mechanism through which social skills training influences children’s cognitive concentration by highlighting the crucial mediating role played by their SIP skills.
2. The SIP skills are crucial for enhancing children’s cognitive concentration in social skills training programs. Although the direct effect of LBF intervention on children’s cognitive concentration is not significant, it can significantly influence their cognitive concentration through encoding and hostile attribution in SIP skills. Therefore, the SIP skills play a fully mediating role between LBF intervention and children’s cognitive concentration. In other words, by reducing children’s sensitivity to hostile cues and minimizing the likelihood of making hostile attributions (For example, children with cognitive impairments may be more inclined to interpret classroom information as exclusionary or isolating, and as a result, they may exhibit learning fatigue, inattentiveness, and lack of effort.), social skills training effectively enhances cognitive concentration. This research finding further validates the accuracy and significance of the SIP theory in interventions aimed at reducing aggression in children, providing robust support for its development and application.
3. Social skills training influences gender differences in cognitive concentration through children’s SIP skills. Crick and Dodge (1996) classified aggressive behavior into reactive aggression and proactive aggression, suggesting that children with reactive aggression are more likely to exhibit hostile biases when interpreting cues and attributing intentions of peers, while children with proactive aggression tend to evaluate aggressive behavior and their consequences more positively. This study further identified gender differences within this classification, indicating that social skills training significantly enhances girls’ skills to interpret cues and subsequently improves their cognitive concentration by effectively reducing reactive aggression. Simultaneously, the intervention has a significant impact on decreasing boys’ hostile attribution, enhancing their response decision level, and improving their cognitive concentration by effectively reducing both reactive and proactive aggression. In the preliminary research of the “LBF (Shaanxi)” program, a random effects model for cognitive concentration revealed that boys had a significantly lower level compared to girls by 1.423 units, demonstrating a notable gender difference. Furthermore, this study found that social skills training is more effective in improving boys’ cognitive concentration than girls’, as evidenced by standardized path coefficients and significant paths quantity analysis. Therefore, it can be concluded that social skills training programs based on SIP theory are particularly important for enhancing boys’ cognitive concentration. This finding also supports Lansford et al.’s (2006) conclusion that aggressive behavior is more prevalent among boys than girls; thus interventions are especially crucial for boys.

Furthermore, cognitive concentration primarily represents children’s adaptability in the classroom, encompassing numerous variables related to academic achievement that are not present in other aggressive behavior. Therefore, it is possible to enhance children’s cognitive concentration by implementing the LBF program designed for 8 to 10-year-olds. By improving children’s cognitive concentration, their academic achievement can be enhanced through their own efforts rather than relying on factors such as environment or genetics that are difficult to change.

## 7 Limitations

This study has some areas for improvement, particularly regarding low factor loadings in variable measurement. In future measurements, adjustments can be made based on the Chinese context to enhance measurement validity. Another limitation of this study is the exclusion of children’s academic performance from the model; further research can explore this aspect.

## Data Availability

The availability of the data is restricted due to privacy or ethical considerations. Requests to access the datasets should be directed to the corresponding author.

## Footnotes

^1^Sample size of each school. LBF group: School 1 = 20, School 2 = 32, School 3 = 27, School 4 = 28, School 5 = 33, School 6 = 15, School 7 = 42, School 8 = 17, School 9 = 35, School 10 = 44, School 11 = 19, School 12 = 12, School 13 = 19 Control group: School 14 = 25, School 15 = 28, School 16 = 28, School 17 = 37, School 18 = 30, School 19 = 29, School 20 = 27, School 21 = 13, School 22 = 31, School 23 = 33, School 24 = 9, School 25 = 11, School 26 = 12, School 27 = 25.
